# Application of urine immunofixation electrophoresis in prognostic evaluation of hematopoietic stem cell transplantation in patients with myeloma

**DOI:** 10.12669/pjms.38.1.4425

**Published:** 2022

**Authors:** Shanshan Zhu, Chao Yang, Wei Li, Meilin Lin

**Affiliations:** 1 Shanshan Zhu, Clinical Laboratory, Affiliated Hospital of Hebei University, Baoding, 071000, Hebei, China; 2 Chao Yang, Clinical Laboratory, Affiliated Hospital of Hebei University, Baoding, 071000, Hebei, China; 3 Wei Li, Clinical Laboratory, Affiliated Hospital of Hebei University, Baoding, 071000, Hebei, China; 4 Meilin Lin, Clinical Laboratory, Affiliated Hospital of Hebei University, Baoding, 071000, Hebei, China

**Keywords:** Hematopoietic stem cell transplantation, Immunofixation electrophoresis, Multiple myeloma, Prognosis, Urine

## Abstract

**Objectives:**

To investigate the value of urine immunofixation electrophoresis in prognostic evaluation of hematopoietic stem cell transplantation in patients with myeloma.

**Methods:**

Thirty-four patients with multiple myeloma admitted to Affiliated Hospital of Hebei University from November 2013 to December 2014 were included as research subjects. All patients received hematopoietic stem cell transplantation and were followed up for five years. Outcomes were evaluated according to the recovery status: complete response (CR), very good partial response (VGPR), partial response (PR), stable disease (SD), and progression disease (PD). In addition, the overall response rate (CR+VGPR) of patients was observed and their urine immunoglobulin status was measured by immunofixation electrophoresis. The Kaplan-Meier method was utilized to plot the survival curve, and the Log-rank method was adopted to analyze the relationship between CR+VGPR and PR and hematopoietic stem cell transplantation (HSCT) survival in patients with myeloma.

**Results:**

The basic clinical type of immunofixation electrophoresis was as follows: 19 cases (55.88%) of IgG, 7 cases (20.59%) of IgA, 6 cases (17.65%) of IgM, and 2 cases (5.88%) of light chain type. Outcomes: 13 cases (38.24%) of CR, 12 cases (35.29%) of VGPR, 9 cases (26.47%) of PR, and 25 cases (73.53%) of the overall response rate (CR+VGPR). Compared with IgG, CR, VGPR and PR of IgA, IgM and light chain had statistically significant differences in outcome (*p*<0.05), and CR+VGPR of patients with IgG was higher than that of patients with IgA, IgM and light chain type (*p*<0.05). Two of the 34 patients were lost to follow-up. The log-rank analysis showed that the survival rate of patients with CR+VGPR was higher than that of patients with PR (*p*<0.05). Patients with IgA, IgM, and light chain type had an increased number of prognostic death compared with those with IgG (*p*<0.05).

**Conclusion:**

Patients with IgG type myeloma are superior to those with IgA, IgM and light chain type in terms of the prognosis of hematopoietic stem cell transplantation, which has a certain clinical reference value.

## INTRODUCTION

Multiple myeloma is a malignant proliferation of plasma cells in bone marrow in which clonal plasma cells in the bone marrow proliferate abnormally and secrete monoclonal immunoglobulins or fragments thereof, causing damage to the corresponding organs or tissues and exacerbating the disease process. Multiple myeloma is clinically manifested as bone pain, localized mass, sepsis, hemocytopenia or leukopenia.^1^,^2^ Various approaches, such as autologous hematopoietic stem cell transplantation, second-generation proteasome inhibitors, immunomodulators, and monoclonal antibodies targeting surface proteins, have been used in the treatment of multiple myeloma.^3^ With these approaches, the prognosis of patients can be greatly improved, some patients respond poorly to treatment, and almost all patients will relapse.^4^ Therefore, it is of great importance to explore the factors affecting the prognosis and to carry out an early intervention. Immunofixation electrophoresis boasts a certain application value in the detection of plasma cell disease^5^, and has a certain diagnostic value in the diagnosis of multiple myeloma, with high sensitivity and obvious resolution.^6^,^7^ However, this technique has been less studied in the prognosis of disease treatment. Consequently, in this study, urine immunofixation electrophoresis was utilized to detect Ig typing in patients with myeloma after hematopoietic stem cell transplantation, so as to explore the relationship between Ig typing and prognosis. The specific details are now reporting as following.

## METHODS

Thirty-four patients with multiple myeloma admitted to Affiliated Hospital of Hebei University from November 2013 to December 2014 were collected as research subjects. Among them, there were 21 males and 13 females, aged 47-63 years old, with an average age of (54.69±4.46) years old. The diagnostic criteria for patients with myeloma are in line with the diagnostic criteria of the “Guidelines for the Diagnosis and Management of Multiple Myeloma in China” (2013 Revision) 8.

### Ethical Approval:

The study was approved by the Institutional Ethics Committee of Affiliated Hospital of Hebei University(No.20190924; date: April 2018), and written informed consent was obtained from all participants

### Inclusion criteria:

- Patients meeting the above diagnostic criteria;
- Patients who were newly diagnosed and untreated;
- Patients with normal mental health;
- Patients aged ≥ 18 years.

### Exclusion criteria:

- Patients who had received radiotherapy or chemotherapy;
- Patients with a history of drug allergy;
- Patients complicated with cardiovascular diseases;
- Female patients in pregnancy and lactation period.

### Prophase treatment of hematopoietic stem cell transplantation:

All patients received chemotherapy after admission. According to the actual condition of the patients, 19 of the 34 patients were treated with vincristine (Shenzhen Wanle Pharmaceutical Co., Ltd., State Drug Approval No.: H44021772) plus adriamycin (Jiaxing Aisen Chemical Co., Ltd., CAS No.: 23214-92-8) plus dexamethasone (Beijing Front Pharmaceutical Co., Ltd., State Drug Approval No.: H34022251) plus thalidomide (Changzhou Pharmaceutical Factory Co., Ltd., State Drug Approval No.: H32026128) for chemotherapy. The remaining 15 cases were treated with bortezomib (Xi’an Janssen Pharmaceutical Co., Ltd., State Drug Approval No.: H20170551).

Chemotherapy combined with recombinant human granulocyte colony stimulating factor (rhG-CSF) was utilized to mobilize peripheral blood hematopoietic stem cell transplantation. Protocol reference^9^: Phosphamide 2-39/m2/d×2d, etoposid-16 500mg×2d. After chemotherapy, patients were treated with 300μg/d of rhG-CSF after peripheral white blood cell count < 1×109/L. When the white blood cell count > 4×109/L and the platelet count > 50×109/L, hematopoietic stem cells were collected by a blood cell separator (Terumo BCT, Article No.: Elutra) for 2d, with a circulating blood volume of 8000mL-12000ml for each collection. The hematopoietic stem cell suspension was added with an equal volume of protective solution (plasma and xylene sulphoxide: 10% of the final concentration), and stored in liquid nitrogen at -196°C after gradient freezing. The suspension was quickly thawed in a water bath at 40°C for reinfusion, and then the hematopoietic stem cells were reinfused.

### Pretreatment regimen and post-transplant treatment:

All patients received hematopoietic stem cell transplantation. According to the actual conditions of the patients, 22 of the 34 patients received 200mg/m2 melphalan (Gleevec) (Jiangsu Chia Tai-Tianqing Pharmaceutical Co., Ltd., State Drug Approval No.: H20174152) after treatment; eight cases were treated with 160mg/m2 melphalan plus 1000mg etoposid-16 plus carmustine (Hebei Meitu Pharmaceutical Co., Ltd., State Drug Approval No.: H13023026); The remaining four cases were treated with 140mg/m^2^ melphalan.

After treatment, 100mL of morning urine was collected and centrifuged at 3000rpm for five minues to filter all cells and tissue fragments. With immobilized immunoelectrophoresis, a fully automated rapid electrophoresis analysis system (Helena, Inc., USA, Model: Helena) and associated reagents were used for electrophoresis and scanning, and a rate scattering turbidimetric method was adopted to determine proteins. The protein electrophoresis profile (ELP) was used as a reference lane to show the electrophoretic proteins, and the remaining five lanes, namely IgG, IgA, IgM, light chain κ and λ, were used to identify the monoclonal components. All samples showed a narrow, deep staining, and well-defined precipitate, and immunophenotyping was performed on immunofixation electrophoresis based on the M protein.

All patients were followed up for five years by outpatient or telephone. According to the IMWG standard^10^, outcomes can be divided into the following types: complete response (CR), very good partial response (VGPR), partial response (PR), stable disease (SD), and progression disease (PD), and the overall response rate (CR+VGPR) was observed. The number of prognostic survival and prognostic death was calculated based on the 5-year survival status of the patients.

### Statistical Analysis:

All the data were processed with statistical software SPSS25.0 and GraphPad. The measurement data were expressed as [n (%)], and the chi-square test was performed for comparison. The Kaplan-Meier method was utilized to plot the survival curve, and the Log-rank method was adopted to analyze the relationship between CR+VGPR and PR and hematopoietic stem cell transplantation (HSCT) survival in patients with myeloma. P<0.05 indicates a statistically significant difference.

## RESULTS

Basic clinical types: 19 cases (55.88%) of IgG, 7 cases (20.59%) of IgA, 6 cases (17.65%) of IgM, 2 cases (5.88%) of light chain type. Thirty-four patients all received hematopoietic stem cell transplantation, including 13 cases (38.24%) of CR, 12 cases (35.29%) of VGPR, and 9 cases (26.47%) of PR. Among these patients, there were 25 cases (73.53%) of the overall response rate (CR+VGPR).

Differences were found in the relationship between clinical typing and outcome (*p*<0.05). Compared with IgG, CR, VGPR and PR of IgA, IgM and light chain had statistically significant differences in outcome (*p*<0.05), and CR+VGPR of patients with IgG was higher than that of patients with IgA, IgM and light chain type (*p*<0.05). Table-I.

**Table-I:** Relationship between clinical typing and outcome [n (%)].

Clinical typing	Outcome			

	CR (n=13)	VGPR (n=12)	PR (n=9)	CR+VGPR (n=25)
IgG (n=19)	11 (57.89)	8 (42.11)	0 (0.00)	19 (100.00)
IgA (n=7)	2 (28.57)	2 (28.57)	3 (42.86)^a^	4 (57.14)^a^
IgM (n=6)	0 (0.00)	2 (33.33)	4 (66.67)^a^	2 (33.33)^a^
Light chain type (n=2)	0 (0.00)	0 (0.00)	2 (100.00)^a^	0 (0.00)^a^
x^2^	19.689			18.342
p	0.000			0.000

Note:

a p<0.05 compared with IgG.

**Fig.1:**
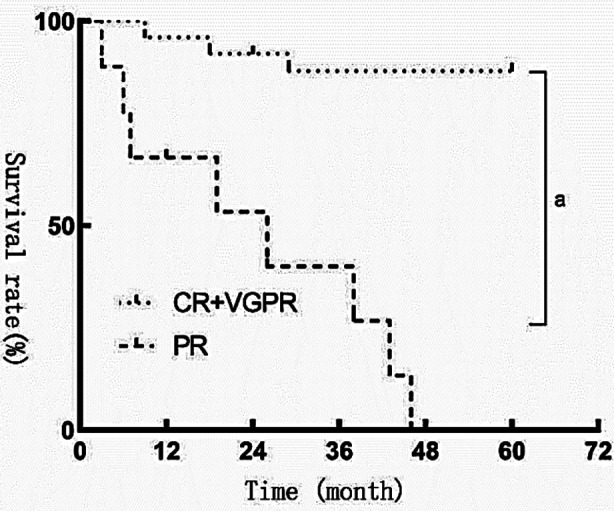
Kaplan-Meier survival curve after hematopoietic stem cell transplantation in myeloma patients with (CR+VGPR) and PR. *Note:* *p*<0.05 compared with (CR+VGPR).

Among the 34 patients, 1 patient with CR+VGPR was dropped to follow-up within 12 months, and 1 patient with PR was dropped to follow-up within 24 months. The survival rate of patients with CR+VGPR was higher than those with PR (log-rank=26.27, *p*=0.000<0.05). Fig.1.Patients with IgA, IgM, and light chain type had an increased number of prognostic death compared with those with IgG (*p*<0.05). Table-II.

**Table-II:** Relationship between the prognosis of hematopoietic stem cell transplantation and immunophenotyping in patients with myeloma [n (%)].

Clinical typing	Prognosis	

	Prognostic survival (n=21)	Prognostic death (n=11)
IgG (n=18)	17 (89.47)	2 (10.53)
IgA (n=6)	3 (50.00)	3 (50.00)^a^
IgM (n=6)	1 (16.67)	5 (83.33)^a^
Light chain type (n=2)	0 (0.00)	2 (100.00)^a^
x^2^	13.724	
p	0.000	

Note:

a p<0.05 compared with IgG.

## DISCUSSION

Multiple myeloma (MM) is still a mystery in terms of its etiology, but severe hazards such as anemia, impaired renal function, impaired nervous system, and in severe cases, paralysis, can be caused to patients with MM.^11^ Patients with MM usually have a median survival time of 3-4 years, and their incidence accounts for about 1% of malignant tumors, showing an increasing trend year by year.^12^ Autologous hematopoietic stem cell transplantation has been extensively applied in the treatment of MM. It is a treatment regimen combined with autologous hematopoietic stem cell therapy based on traditional chemotherapy regimens, such as melphalan plus prednisone, vincristine plus adriamycin plus dexamethasone, which is characterized by addressing the various problems of traditional treatments such as strong dose dependence and bone marrow clearance, and improving the clinical outcome and survival rate of patients.^13^,^14^ Although certain effects can be achieved by autologous hematopoietic stem cell transplantation, IG will have an impact on infections, tumors, and autoimmune diseases, and patients show disparate symptoms due to their Ig types.^15^ Patients with IgG type myeloma are prone to a decline in organism immunity, leading to infection and aggravating the disease progression, those with IgA-type are susceptible to hypercalcemia and hypercholesterolemia, while those with IgM and light chain type are prone to renal dysfunction.^16^-^18^ It may therefore be of definite value to predict the prognosis of HSCT in patients with multiple myeloma based on the IG type of patients.

It can be concluded from this study that M protein appeared in all 34 patients after hematopoietic stem cell transplantation, including 19 cases (55.88%) of IgG, 7 cases (20.59%) of IgA, 6 cases (17.65%) of IgM, and 2 cases (5.88%) of light chain type,. It indicates that the IgG type dominates after hematopoietic stem cell transplantation in patients with MM, followed by IgA and IgM types, and at least the light chain type. The above conclusions are in resemblance with the findings of Zhang et al.^19^ After outcome evaluation, 13 cases (38.24%) of CR, 12 cases (35.29%) of VGPR, 9 cases (26.47%) of PR, and 25 cases (73.53%) of overall response rate (CR+VGPR) are found, indicating that a certain efficacy is achieved after HSCT in patients with MM, with an overall response rate as high as 73.53%. Nevertheless, there are still some patients with poor treatment effects, and the reasons have yet to be further verified.

Differences can be observed in clinical typing and outcome. The CR+VGPR of patients with IgG type is higher than that of patients with IgA, IgM and light chain type, indicating that patients with IgG type have achieved favorable results after the treatment. Further verification of the prognosis, survival and death of patients after treatment show that the median survival time of patients with PR is 26 months, and the survival rate of patients with (CR+VGPR) is higher than that of patients with PR. In this study, all patients with PR died within five years. Patients with (CR+VGPR) have a certain death rate, but it is lower than that of patients with PR. Patients with IgA, IgM, and light chain type had an increased number of prognostic death compared with those with IgG, indicating that patients with IgG type have fewer deaths and better prognosis. In this study, the outcome and survival rate of patients with IgG type are better than those of other types. The prognosis of patients with IgG type is preferable to that of patients with IgM and IgA, and the prognosis of patients with IgM type is the worst. IgG type can affect the immunity of patients. Therefore, it may be beneficial for the prognosis in the course of daily treatment to enhance the immunity of patients, prevent patients from infection and improve the prognosis of patients^20^, and adopt the appropriate treatment regimen on the basis of treatment according to each type.

### Limitations of the study:

The insufficient number of cases leads to a certain bias between the results and the actual situation. In view of this, proactive countermeasures will be taken in order to collect sufficient samples and increase the scope of the study, so as to obtain more accurate study results.

## CONCLUSIONS

Prognosis can be improved by enhancing immunity in patients with IgG type while strictly following the treatment regimen. However, patients with IgA and IgM types may have difficulties in the process of regulation, leading to a poor prognosis. To achieve this corresponding treatment should be adopted in clinical practice according to the corresponding type to improve the prognosis of patients..The outcome and prognostic survival status of patients with IgG type multiple myeloma undergoing hematopoietic stem cell transplantation in urine are better than those of other types, which have certain reference value for clinical prognosis evaluation.

### Authors’ Contributions:

**SZ & CY:** Designed this study, prepared this manuscript, are responsible and accountable for the accuracy, integrity of the work.

**WL:** Collected and analyzed clinical data.

**ML:** Significantly revised this manuscript.
